# Inferring microRNA-Environmental Factor Interactions Based on Multiple Biological Information Fusion

**DOI:** 10.3390/molecules23102439

**Published:** 2018-09-24

**Authors:** Haiqiong Luo, Wei Lan, Qingfeng Chen, Zhiqiang Wang, Zhixian Liu, Xiaofeng Yue, Lingzhi Zhu

**Affiliations:** 1 School of information and management, Guangxi Medical University, Nanning 530021, China; hqluo@163.com; 2 School of Computer, Electronic and Information, Guangxi University, Nanning 530004, China; 3 State Key Laboratory for Conservation and Utilization of Subtropical Agro-bioresources, Guangxi University, Nanning 530004, China; zhqwang@gxu.edu.cn; 4 School of electronic and information engineering, Qinzhou University, Qingzhou 535011, China; qzxylzx@163.com; 5 School of Automation, Huazhong University of Science and Technology, Wuhan 430074, China; xfyue@hust.edu.cn; 6 Department of Computer and Information Science, Hunan Institute of Technology, Hengyang 421008, China; lz_zhu@csu.edu.cn

**Keywords:** microRNA, environmental factor, structure information, similarity network

## Abstract

Accumulated studies have shown that environmental factors (EFs) can regulate the expression of microRNA (miRNA) which is closely associated with several diseases. Therefore, identifying miRNA-EF associations can facilitate the study of diseases. Recently, several computational methods have been proposed to explore miRNA-EF interactions. In this paper, a novel computational method, MEI-BRWMLL, is proposed to uncover the relationship between miRNA and EF. The similarities of miRNA-miRNA are calculated by using miRNA sequence, miRNA-EF interaction, and the similarities of EF-EF are calculated based on the anatomical therapeutic chemical information, chemical structure and miRNA-EF interaction. The similarity network fusion is used to fuse the similarity between miRNA and the similarity between EF, respectively. Further, the multiple-label learning and bi-random walk are employed to identify the association between miRNA and EF. The experimental results show that our method outperforms the state-of-the-art algorithms.

## 1. Introduction

There is increasing evidence demonstrating that phenotypes are associated with genetic factors (GFs) and environmental factors (EFs) [1,2]. Environmental factors, including stress, alcohol, pollution, radiation and drugs play important roles in many diseases [3]. The perturbation of GF-EF interactions may result in some diseases [4,5]. Thus, identifying the potential associations between GFs and EFs is useful for biologists to understand the molecular bases of diseases.

MiRNA is a kind of typical GF with the length from 18 nt to 25 nt. It has been proved that miRNA can regulate the expression of genes by binding to the 3′ untranslated region (UTR) or 5′ untranslated region of mRNA in organisms [6,7]. In addition, accumulated evidence has demonstrated that miRNA normally plays essential roles in many important biological processes, including cell growth, cell cycle control, cell differentiation, cell apoptosis, and so on [8]. Therefore, the functional abnormality of miRNA can cause a broad range of diseases. For example, miR-150 can regulate the expression of the genes GAB1 and FOXP1 and impact the B and T cell activity in chronic lymphocytic leukemia [9]. Recently, a growing number of studies have indicated that miRNAs interact with diverse EFs [10,11,12]. The perturbation of miRNA-EF interactions is also related to a number of human diseases. For example, gemcitabine can down-regulate the expression of hsa-let-7b in pancreatic cancer cells [13,14]. Therefore, identifying potential miRNA-EF interactions contributes to the study of diseases. In addition, with the development of biotechnology, several databases such as miRbase [15], miRecord [16], dbDEMC [17] and miREnvironment [18] have been developed to store miRNA and EF related data. Those databases provide reliable data resources for predicting miRNA-EF interactions.

In recent years, many computational methods have been proposed to predict miRNA-EF interactions [19]. Chen et al. [20] proposed a method called miREFScan based on Laplacian regularized least squares to predict the interactions between miRNAs and EFs. This method is based on the assumption that functionally similar miRNAs tend to be related with similar EFs [21]. Chen et al. [22] presented a computational approach (miREFRWR) to infer miRNA-EF interactions based on a random walk method. Jiang et al. [23] constructed a small molecule-miRNA interaction network in 23 cancers and then identified the miRNA-EF associations based on hypergeometric tests. Qiu et al. [24] revealed several important features of miRNA and EF by analyzing miRNA-EF interaction network and proposed a model based on Fisher tests to infer potential miRNA-EF interactions. Li et al. [25] presented a computational framework based on an EF structure and disease similarity method to predict the interaction. Although the above methods have achieve great successes, some of them use low quality datasets which may result in poor performance. For example, some approaches measure miRNA similarity and EF similarity by using network-based data only, which may result in a bias for ignoring the biological characteristics of miRNA and EF. Most cannot effectively integrate different biological data resources. Further, some methods are unsuitable for predicting interaction of new miRNA without any known related EFs or new EF without any known related miRNAs.

In this paper, we assume that functionally similar miRNAs tend to be related with similar EFs. Based on this assumption, a computational framework is developed to predict the interactions between miRNAs and EFs. Unlike traditional methods, we use different data sources to measure miRNA-miRNA similarity and EF-EF similarity. The former is calculated by using the miRNA sequences and miRNA-EF interaction information, and the EF-EF similarity is computed by the anatomical therapeutic chemical, chemical structure and miRNA-EF interaction information. In particular, the similarity network fusion is applied to integrate these two similarities. Further, the multiple-label learning and bi-random walk are employed to identify the association between miRNA and EF. The experimental results show that our method is effective in inferring miRNA-environmental factor interactions.

## 2. Datasets and Methods

### 2.1. Datasets

We downloaded the known miRNA-EF interaction data from the miREnvironment database (http://www.cuilab.cn/miren) [18], which includes 3857 entries from 24 species. Only the human- related data were used for the following experiments. We manually checked the data and removed the interactions which do not correspond to human diseases. After pruning the invalid information, 224 miRNAs, 124 EFs and 729 miRNA-EF interactions were extracted as the gold dataset. A matrix *I* is constructed to represent miRNA-EF interaction. The value 1 is assigned to *I (i, j)* if the interaction between miRNA *i* and EF *j* can be found, otherwise 0.

miRNA sequence information is obtained from miRbase (version 22) [15], which contains more than 2400 human sequences. After mapping miRNA of the gold dataset to miRbase, 224 miRNA sequences were finally obtained.

We download the chemical structure and anatomical therapeutic chemical of drugs from KEGG database (in 2016) [26]. There are 81 drugs with chemical structure and 57 drugs with anatomical therapeutic chemical, respectively.

### 2.2. Measuring miRNA-miRNA Similarity and EF-EF Similarity

#### 2.2.1. miRNA-miRNA Similarity

Based on assumption that miRNAs with similar function are tend to relate with similar EFs, the interaction profile similarity is utilized to measure the similarity of pairwise miRNAs [27]. The miRNA interaction profile similarity is defined as:

(1) $$\;W_{m}^{p}\left(m_{i},m_{j}\right)=e^{\left(-\gamma_{m}\|IP\left(m_{i}\right)-IP\left(m_{j}\right){\|}^{2}\right)}\;$$

(2) $$\;\gamma_{m}=\frac{1}{\frac{1}{n}\sum_{i=1}^{n}IP\left(m_{i}\right)}\;$$

where $m_{i}$ and $m_{j}$ represent miRNAs *i* and *j*. *n* represents the number of miRNAs. $IP\left(m_{i}\right)$ represents the interactions between miRNA *i* and all EFs in the known miRNA-EF interaction data, *i. e.* the *i*-th row of matrix *I*. The parameter $\gamma_{m}$ is set to control the kernel bandwidth. The sequence information has been widely used to find miRNA-disease association and feature patterns of miRNA regulation inference [28]. The Emboss-needle tool is utilized to compute sequence similarity of pairwise miRNAs [29].

#### 2.2.2. EF-EF Similarity 

The chemical structure is an important piece of information for drug design and has been applied to measure drug similarity [20,30]. SIMCOMP [31] is used to calculate the similarity of pairwise drugs based on common substructures. In addition, the Anatomical Therapeutic Chemical (ATC) code obtained from the ATC Classification System [26] assists in calculating the pairwise similarity of drugs.

Based on the assumption that EFs with similar function are tend to relate with similar miRNA, the interaction profile similarity is employed to measure the similarity between EFs [27]. The EF interaction profile similarity is defined as:

(3) $$\;W_{e}^{p}\left(e_{i},e_{j}\right)=e^{\left(-\gamma_{e}\|IP\left(e_{i}\right)-IP\left(e_{j}\right){\|}^{2}\right)}\;$$

(4) $$\;\gamma_{e}=\frac{1}{\frac{1}{m}\sum_{i=1}^{m}IP\left(e_{i}\right)}\;$$

where $e_{i}$ and $e_{j}$ represent EFs *i* and *j*. *m* denotes the number of EFs. $IP\left(e_{i}\right)\;$represents the interaction between EF *i* and all miRNAs in the known miRNA-EF interaction data*, i. e.* the *i*-th column of matrix *I*. The parameter $\gamma_{e}$ is to control the kernel bandwidth.

### 2.3. Similarity Network Fusion

The similarity network fusion (SNF) is an approach for multiple omics fusion, which has been widely used for cancer data analysis [32,33]. It is able to capture the global and local features of different data. The SNF for miRNA is defined as follows:

(5) $$\;F^{m}=\frac{F_{m}^{s}+F_{m}^{p}}{2}\;$$

(6) $$\;F_{m}^{p}\left(t\right)=L_{m}^{p}\times G_{m}^{s}\left(t-1\right)\times {\left(L_{m}^{p}\right)}^{T}\;$$

(7) $$\;F_{m}^{s}\left(t\right)=L_{m}^{s}\times G_{m}^{p}\left(t-1\right)\times {\left(L_{m}^{s}\right)}^{T}\;$$

(8) $$\;L_{m}^{s}\left(i,j\right)=\{\begin{array}{c} \frac{W_{m}^{s}\left(i,j\right)}{\sum_{k\in N_{i}}W_{m}^{s}\left(i,k\right)},\;j\in N_{i}\\ 0,\;\;otherwise \end{array}\;$$

(9) $$\;L_{m}^{p}\left(i,j\right)=\{\begin{array}{c} \frac{W_{m}^{p}\left(i,j\right)}{\sum_{k\in N_{i}}W_{m}^{p}\left(i,k\right)},\;j\in N_{i}\\ 0,\;\;otherwise \end{array}\;$$

(10) $$\;G_{m}^{s}\left(i,j\right)=\{\begin{array}{c} \frac{W_{m}^{s}\left(i,j\right)}{2\sum_{k\neq i}W_{m}^{s}\left(i,k\right)},\;i\neq j\\ \frac{1}{2},\;\;i=j \end{array}\;$$

(11) $$\;G_{m}^{p}\left(i,j\right)=\{\begin{array}{c} \frac{W_{m}^{p}\left(i,j\right)}{2\sum_{k\neq i}W_{m}^{p}\left(i,k\right)},\;i\neq j\\ \frac{1}{2},\;\;i=j \end{array}\;$$

where $W_{m}^{s}\;$and $W_{m}^{p}\;$denote the miRNA sequence similarity matrix and miRNA interaction profile similarity matrix, respectively. $G_{m}^{s}$, $L_{m}^{s}$*,* $G_{m}^{p}$ and $L_{m}^{p}$ denote the global matrix of miRNA sequence similarity, local matrix of miRNA sequence similarity, global matrix of miRNA interaction profile similarity, local matrix of miRNA interaction profile similarity, respectively. The *N_i_* represents the *K*-nearest neighbors of miRNA *i*. $F_{m}^{s}$ and $F_{m}^{p}$ denote the fusional matrix of miRNA sequence similarity and the fusional matrix of miRNA interaction profile similarity, respectively. $F^{m}$ denotes the final fusional matrix of miRNA. The final fusional matrix of EF $F^{e}$ can be obtained in term of similar manner.

### 2.4. Inferring miRNA-EF Interaction by Using bi-Random Walk and Multi-Label Learning (MEI-BRWMLL)

Considering the features of bi-random walk and multi-label learning, we utilize a bi-random walk to infer interactions of known miRNA/EF and multi-label learning is used to infer interactions of new miRNA/EF. The reason for selecting these two methods is that the bi-random walk achieves good results in potential interaction prediction between known entities while multi-label learning is robust in predicting interactions between new entities. 

#### 2.4.1. Bi-Random Walk for Predicting Potential Interactions of Known miRNAs and EFs

Based on assumption that similar miRNAs tend to relate with similar EF, the bi-random walk is employed to predict potential miRNA-EF interaction. 

Firstly, the miRNA similarity matrix and EF similarity matrix are normalized by using Laplace regularization, respectively. It is defined as:

(12) $$\;N^{m}=D^{m}{}^{-\frac{1}{2}}\times F^{m}\times D^{m}{}^{-\frac{1}{2}}\;$$

(13) $$\;N^{e}=D^{e}{}^{-\frac{1}{2}}\times F^{e}\times D^{e}{}^{-\frac{1}{2}}\;$$

where *N^m^* and *N^e^* represent normalized matrix of fusional miRNA similarity and EF similarity, respectively. *D^m^* and *D^e^* represent the diagonal matrix of *F^m^* and *F^e^*, respectively. In addition, the miRNA-EF interaction matrix *I* is normalized as follows:

(14) $$\;N^{I}\left(i,j\right)=\frac{I\left(i,j\right)}{\sum_{i}\sum_{j}I\left(i,j\right)}\;$$

Then, we use bi-random walk to predict potential miRNA-EF interaction by walking on miRNA similarity network and EF similarity network. The iterative process of bi-random walk is defined as follows:

Left walk in miRNA similarity network:

(15) $$\;R_{L}\left(t\right)=\alpha \times N^{m}\times R_{L}\left(t-1\right)+\left(1-\alpha \right)\times N^{I}\;$$

Right walk in EF similarity network:

(16) $$\;R_{R}\left(t\right)=\alpha \times R_{R}\left(t-1\right)\times N^{e}+\left(1-\alpha \right)\times N^{I}\;$$

The final predicted score is defined as follows:

(17) $$\;R\left(t\right)=\frac{R_{L}\left(t\right)+R_{R}\left(t\right)}{2}\;$$

where *R_L_(t)* and *R_R_(t)* denote the predicted score matrix of walk on miRNA similarity network and EF similarity network at step *t*, respectively. *R(t)* denotes the final score matrix at step *t*. In addition, the miRNA similarity network and EF similarity network contain different topological and structural features, and the optimal iteration steps of the random walk on the two networks should be different. Therefore, we set two parameters *l*, *r* to control the maximal random walk steps on two networks, respectively. The iterative of bi-random walk will stop when the number of iteration *t* exceeds the maximum of parameters *l* and *r*. The parameters can accelerate the iteration termination. In here, the *l* and *r* are set as 4 and 2, respectively.

#### 2.4.2. Multi-Label Learning for Predicting Interactions of New miRNAs and EFs

We employ multi-label learning to infer the interactions of new miRNA/EF, which predicts the label of unseen instances based on a maximum a posteriori rule [34,35]. For convenience, we define some notations. miRNAs and EFs are assigned two domains *D_M_* = {*m_1_,m_2_,…m_x_*} and *D_E_* = {*e_1_,e_2_,…e_y_*}, respectively. *x* and *y* represent the numbers of miRNAs and EFs, respectively. The interactions between miRNAs and EFs are represented by matrix $I_{x\times y}$. *P_ij_* denotes the interaction probability of miRNA *m_i_* and EF *e_j_*. *P_ij_* is set to 1 if *I(i,j) =* 1; otherwise, 0. For a new miRNA *m_c_*, the probability *P(m_c_,e_j_)* between *m_c_* and EF *e_j_* demonstrates the confidence that miRNA *m_c_* is linked to EF *e_j_*. Based on the similarity of miRNA-miRNA, we select the *k* nearest neighbors of miRNA *m_c_*. Then, the probability *P(m_c_,e_j_)* is calculated as follows:

(18) $$\;P\left(m_{c},e_{j}\right)=\frac{P\left(L_{1}^{j}\right)P\left(E_{s}^{j}|L_{1}^{j}\right)}{P\left(L_{1}^{j}\right)P\left(E_{s}^{j}|L_{1}^{j}\right)+P\left(L_{0}^{j}\right)P\left(E_{s}^{j}|L_{0}^{j}\right)}\;$$

(19) $$\;P\left(L_{1}^{j}\right)=\frac{1+\sum_{i=1}^{x}I\left(i,j\right)}{2+x}\;$$

(20) $$\;P\left(L_{0}^{j}\right)=1-P\left(L_{1}^{j}\right)\;$$

(21) $$\;P\left(E_{s}^{j}|L_{1}^{j}\right)=\frac{1+e\left(s\right)}{k+1+\sum_{i=0}^{k}e\left(i\right)}\;$$

(22) $$\;P\left(E_{s}^{j}|L_{0}^{j}\right)=\frac{1+e^{\prime }\left(s\right)}{k+1+\sum_{i=0}^{k}e^{\prime }\left(i\right)}\;$$

where *k* represents the number of nearest neighbors. *e(s)* represents the number of miRNA related to EF *e_j_* whose KNNs contain exactly *s* miRNAs related EF *e_j_*. *e’(s)* counts the number of miRNA unrelated to EF *e_j_* whose KNNs contain exactly *s* miRNAs related EF *e_j_*.

**Figure 1:**
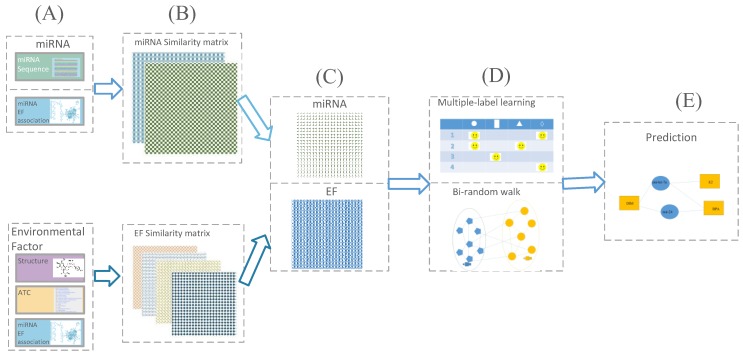
The flowchart of miRNA-EF interaction prediction. (**A**) Computing similarities of miRNA-miRNA and EF-EF, respectively. (**B**) Establishing similarity matrices of miRNA and EF, respectively. (**C**) Integrating similarity matrices of miRNA-miRNA and EF-EF by using similarity network fusion method, respectively. (**D**) Predicting miRNA-EF interactions by using multi-label learning and bi-random walk. (**E**) The final predicted results.

The flowchart for miRNA-EF interaction prediction is shown in Figure 1. Firstly, the similarities of miRNA and EF are calculated based on different similarity measures, respectively. Secondly, the similarity matrices of miRNA and EF are constructed in terms of similarity scores calculated previously. Further, the similarity network fusion is employed to integrating different similarity matrices of miRNA and EF, respectively. Finally, the bi-random walk and multi-label learning are used to infer potential miRNA-EF interactions.

## 3. Experiments

### 3.1. Analyzing the miRNA-EF Interaction Network

**Figure 2:**
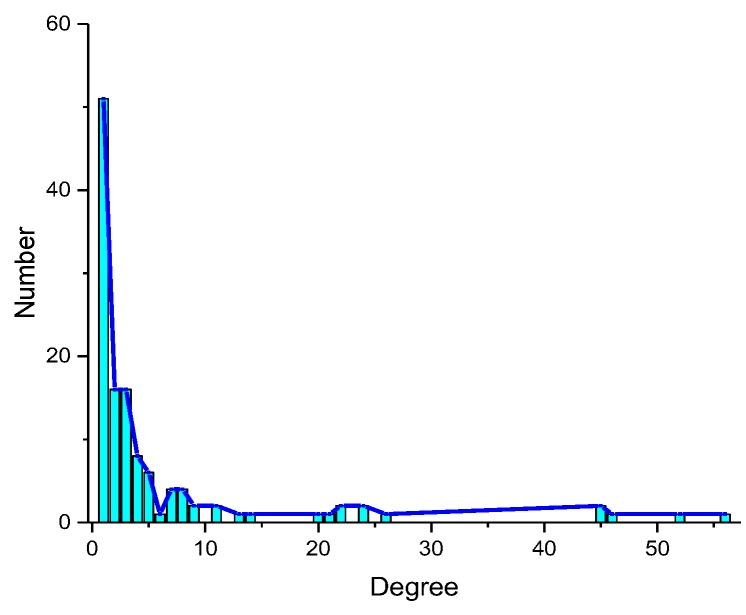
The degree of EFs.

There are 729 interactions between 224 miRNAs and 124 EFs in the whole miRNA-EF interaction network. The degree of EFs is shown in Figure 2. It is observed that the degree of most EFs is equal to 1. It means that most of EFs only have one related miRNA and a great amount of interactions are still unknown. The EF with the max degree is gemcitabine which has 56 related miRNAs. 

**Figure 3:**
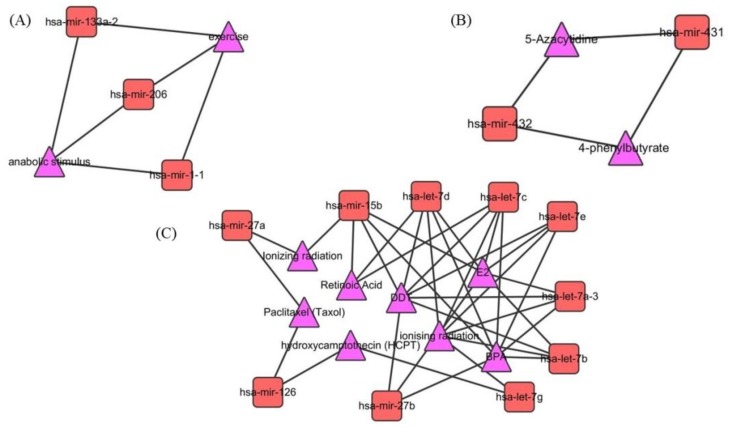
Three modules are obtained from miRNA-EF interaction network by utilizing ClusterViz. (**A**) The EFs (anabolic stimulus and exercise) are related with hsa-mir-133a-2, hsa-mir-206 and hsa-mir-1-1. (**B**) The EFs (5-Azacytidine and 4-phenylbutyrate) are associated with hsa-mir-431 and hsa-mir-432. (**C**) The EFs (DDT, E2, BPA and ionizing radiation) have associations with the let-7 family.

In order to analyze the cluster feature of miRNA-EF interaction network, the ClusterViz [36] program is used to obtain clusters from the network. In Figure 3, three modules are obtained from the miRNA-EF interaction network. This demonstrates that EFs can regulate a group of functionally similar miRNAs rather than a single miRNA. Take the module (C) for example, it demonstrates that four EFs (DDT, E2, BPA and ionizing radiation) have associations with the let-7 family.

### 3.2. Experiment

**Figure 4:**
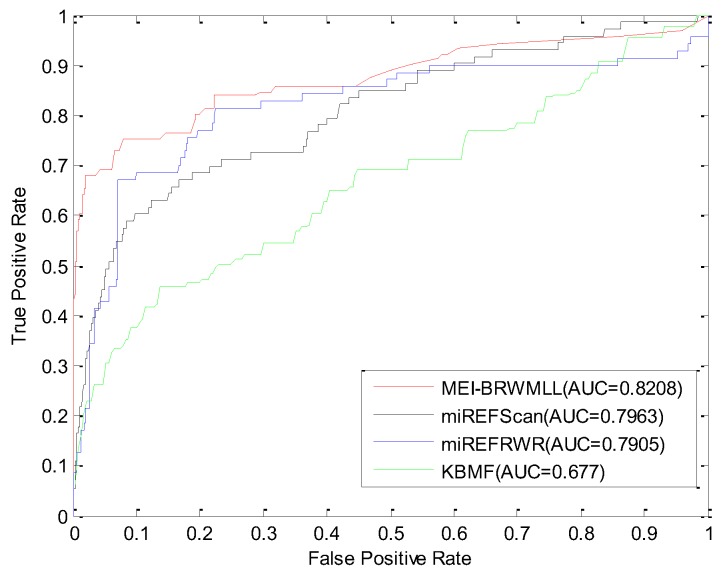
Comparison of different methods in miRNA-EF interaction prediction.

To demonstrate the effectiveness of our method, a comparison between our method and three state-of-the-art methods (miREFScan [20], miREFRWR [22] and KBMF [6]) is conducted. The parameters of these methods are specified as the default value. The 10-fold cross validation is utilized to evaluate the performance of different methods. The known miRNA-EF interactions are divided into 10 subsets. One subset is used as test set and the remaining nine subsets are treated as training set. Then, the true positive rates (TPR) and false positive rates (FPR) are calculated by using different classification thresholds. The receiver operating characteristics (ROC) curve is drawn based on the value of TPR and FPR and the area under the ROC curve (AUC) is calculated to measure the performance. The higher of AUC value, the better performance is. The experimental result is shown in Figure 4. It can be found that our method achieves an AUC of 0.8208 which is better than other two methods (miREFRWR: 0.7905, miREFScan: 0.7963 and KBMF: 0.677). 

### 3.3. Case Study

3,3′-Diindolylmethane (DIM) is a kind of compound widely found in *Brassica* vegetables [37]. An increasing number of studies have shown that DIM has a close relationship with many cancers. For example, it has been proved that the expression of HDAC1 can be inhibited by DIM in colon cancer tissue [38]. Table 1 shows the top 15 potential miRNAs related with DIM which are identified by using MEI-BRWMLL nine miRNAs are confirmed to connect to DIM by the recent literature. It has been proved that the expression of hsa-mir-146a (ranked at first) is induced by DIM in pancreatic cancer cells [39]. In addition, the DIM has been certified to up-regulate miRNA-16 (ranked second) in CD4+ T cells [40]. The literature shows DIM has relationship with hsa-mir-181d, hsa-mir-125b and hsa-mir-34a (ranked at 6th, 8th and 12th), respectively [41,42]. DIM can inhibit the expression of these three miRNAs in SEB-mediated liver injury. The hsa-mir-200b (ranked at 9th) is upregulated by DIM in SKBR3 breast cancer cells [43]. It has been proved that the expression of hsa-mir-221 (ranked at 11th) can be downregulated in pancreatic cancer [44]. The DIM can inhibit the expression of EZH2 by up-regulating hsa-let-7e (ranked at 13th) in castration-resistant prostate cancer [45]. The literature [43] shows that the expression of hsa-mir-200c is up-regulated by DIM and herceptin in breast cancer. In addition, it can be found that several miRNAs are identified to be related with DIM. However, the functions of these miRNAs are still unknown. This requires biologists to validate them by using biological experiments.

## 4. Conclusions

Understanding the complex pathogenesis of diseases is still a significant challenge in disease research [46,47]. Increasing studies have demonstrated that diseases have close relationship with GFs and EFs [48,49]. miRNAs are a group of important GFs which have been proved to play critical roles in many diseases [50,51]. Therefore, identifying miRNA-EF interactions is helpful for elucidating the pathogenesis of diseases. In this paper, a computational framework to predict interactions between miRNAs and EFs is proposed. Multiple biological data are used to measure the pairwise similarity of miRNA-miRNA and EF-EF, respectively. Then, the similarities of miRNA-miRNA and EF-EF are fused by using SNF, respectively. Further, the bi-random walk and multiple label learning are utilized to infer miRNA-EF interactions. The experimental results show that this method is effective for miRNA-EF interaction identification.

## Figures and Tables

**Table 1:** The top 15 potential miRNAs related to 3,3′-diindolylmethane predicted by MEI-BRWMLL.

Rank	miRNA	Evidence
1	hsa-mir-146 a	PMID: 20124483
2	hsa-mir-16	PMID: 24899890
3	hsa-mir-24	Unknown
4	hsa-mir-155	Unknown
5	hsa-mir-223	Unknown
6	hsa-mir-181 d	PMID: 25706292
7	hsa-mir-181 b	Unknown
8	hsa-mir-125 b	PMID: 25706292
9	hsa-mir-200 b	PMID: 23372748
10	hsa-mir-126	Unknown
11	hsa-mir-221	PMID: 24224124
12	hsa-mir-34 a	PMID: 25706292
13	hsa-let-7 e	PMID: 22442719
14	hsa-mir-200 c	PMID:23372748
15	hsa-mir-222	Unknown
